# Aquatic omnivores shift their trophic position towards increased plant consumption as plant stoichiometry becomes more similar to their body stoichiometry

**DOI:** 10.1371/journal.pone.0204116

**Published:** 2018-09-20

**Authors:** Peiyu Zhang, Reinier F. van den Berg, Casper H. A. van Leeuwen, Brigitte A. Blonk, Elisabeth S. Bakker

**Affiliations:** Department of Aquatic Ecology, Netherlands Institute of Ecology (NIOO-KNAW), Wageningen, The Netherlands; University of Connecticut, UNITED STATES

## Abstract

Human induced eutrophication has strongly altered aquatic ecosystems. With increasing eutrophication, plant nutrient concentrations increase, making them more attractive as food for herbivores. However, most aquatic consumers are omnivorous. Ecological stoichiometry theory predicts that animals prefer to consume food which has a similar nutrient (N and P) composition or C:nutrient ratio compared to their own bodies, hence omnivorous animals may prefer to eat animal prey instead of plants. We asked whether aquatic omnivores would shift their diet towards more plant consumption when plants are more nutritious and their stoichiometry becomes more similar to the stoichiometry of the omnivore. We hypothesized that: (1) the omnivore increases plant consumption as the plant C:nutrient ratio decreases when there is only plant material available; (2) the omnivore generally prefers animal food over plant material; (3) the omnivore will increase its relative plant consumption as the plant C:nutrient ratio decreases, in the presence of animal food. As a model system, we used the pond snail *Lymnaea stagnalis* (omnivorous consumer), the aquatic plant *Potamogeton lucens* (plant food to the consumer, cultured at different nutrient regimes to obtain different plant C:nutrient ratios), and the crustacean *Gammarus pulex* (animal food to the consumer, using freshly dead individuals). When there was only plant material available, the consumers increased their relative consumption rate with decreasing plant C:nutrient ratio from no measurable amount to about 102 mg g^-1^ day^-1^. When plant material was offered simultaneously with animal food, even though the omnivores always preferred animal food over plant material, the omnivores still increased their relative intake of plant material as plant C:nutrient ratio decreased, from virtually nothing at the highest to on average 16% of their diet at the lowest plant C:nutrient ratio, with a maximum of 28%. Therefore, we conclude that as nutrient loading increases in aquatic ecosystems, plant-eating omnivorous animals may shift their trophic position towards increased plant consumption and alter the food web structure. As a result, we may observe increased top-down control on aquatic plants.

## Introduction

Nutrient loading caused by anthropogenic activities has strongly altered the structure and functioning of aquatic ecosystems [1–3]. In shallow aquatic systems, aquatic plant communities are important components because they stabilise clear water states [4] and sustain high biodiversity [5, 6]. Submerged aquatic plant communities have rapidly declined because of eutrophication [7, 8]. The classical underlying mechanism for rapid plant declines is the fast growth of algae that outcompete plants at high nutrient loadings [1, 9]. However, more recent insights also point to increased herbivory on aquatic plants as a reason for their decline, as herbivores can have a large impact on aquatic plants [10, 11].

More specifically, it has been hypothesized that the impact of herbivores on aquatic plants increases as plant quality increases [12, 13]. The underlying mechanism is that, when plant nutrient concentration increases with nutrient loading to a water body, these plants would be more attractive to herbivores, experience more grazing, resulting in enhanced top-down control under eutrophic conditions [13]. However, most aquatic plant-consuming animals are omnivores [10, 14], which means that they feed on both plant and animal material. Omnivores can actively select preferred food types if both types are available. There are many factors which will govern the diet selection by omnivores, such as omnivorous nutrient demand, food nutrient content, food availability and defence mechanisms (secondary metabolites in plants) [15, 16]. Ecological stoichiometry theory predicts that animals prefer to consume food which has similar nutrient (N and P) composition or C:nutrient ratio compared to their own bodies [17, 18]. Animal prey has a more similar C:nutrient ratio with omnivores compared to plant material, which generally has much higher C:nutrient ratio than its animal consumers [17, 19]. Hence, according to ecological stoichiometry theory, omnivorous animals would generally prefer animal prey over plant material. In this scenario plant material would only be eaten, when not enough animal material is available [20, 21]. However, we hypothesize that this may change when the stoichiometry of plant material becomes more similar to the stoichiometry of animal prey.

Whereas animal food has a more stable stoichiometric composition than plants [18, 22, 23], plants are more flexible, meaning that their quality as food for consumers may increase as a result of eutrophication. If plants become more nutrient rich under eutrophic conditions, this could decrease the Carbon: nutrient ratio gap between plant material and animal food. This could be an underlying mechanism explaining patterns of selective foraging such as previously found in grazing experiments with ducks [12]. Indeed, aquatic animals prefer plants with a higher nitrogen concentration and lower C:N ratio [21, 24]. Furthermore, studies have shown that omnivorous animals shift their trophic position towards more plant consumption upon eutrophication in terrestrial omnivores [25] and omnivorous marine plankton [26]. However, there are no studies to date which have directly tested the effects of intraspecific variation in plant quality on plant consumption by aquatic omnivores. The consequences of eutrophication for the impact of omnivorous animals on aquatic plants remain therefore largely unknown for aquatic ecosystems.

Here we use an aquatic plant-animal prey-omnivore system to experimentally test the consumption rates of omnivorous aquatic animals in response to changing plant quality, expressed as nutrient concentration or C:nutrient ratio, in the presence and absence of animal food. We hypothesise that: (1) the omnivore increases plant consumption as plant quality increases when there is only plant material available; (2) the omnivore generally prefers animal food over plant material; (3) the omnivore will increase its relative plant consumption as plant quality increases, in the presence of animal food.

## Methods and materials

### Model omnivore

We used the generalist omnivore *Lymnaea stagnalis* L., the great pond snail, because it represents a common, generalist omnivorous consumer in aquatic systems and aquatic molluscs can have large impacts on aquatic plant abundance [11, 27, 28]. *L*. *stagnalis* has been previously used for plant feeding trials in aquatic settings [24, 29–31]. *L*. *stagnalis* feeds on vascular plants [32, 33] as well as carrion, such as dead insects, crayfish, frog tadpoles, fish and even other dead snails [34]. It can distinguish high and low quality food by perception of volatile organic compounds released by the food [35].

Egg clusters from captive *L*. *stagnalis* were collected in a pond on the terrain of NIOO-KNAW, Wageningen, The Netherlands (51°59'14.8"N, 5°40'14.8"E) and hatched, after two weeks the juveniles were transferred to square plastic buckets (0.38 × 0.26 × 0.27 m, L × W × H), each filled with 15 liters of groundwater, and exposed to a 16 : 8 h day : night cycle at a constant temperature of 20°C. Snails were reared on a diet of butterhead lettuce five days per week, and fish food pellets (Velda, Gold Sticks Basic Food) and chalk (ensured sufficient calcium for shell development) were supplied once a week to provide other nutrients. Culturing water was replaced weekly and constantly aerated. All snails were cultured for over 100 days before performing the feeding trial. Snails used in the trials had an average shell length of 30.2 ± 2.4 mm (mean ± SD, n = 94).

### Plant food

*Potamogeton lucens* L. was chosen as the plant material, as it is a common native plant in The Netherlands and one of the most preferred submerged aquatic plants by *L*. *stagnalis* [24, 33]. *P*. *lucens* rhizomes were collected from a ditch to the west of Wageningen, The Netherlands (51.966484°N, 5.620158°E). To obtain plant material of varying nutrient contents, 76 individual rhizomes were planted individually in 76 square bins (20.5 × 19 × 27cm) and placed in 19 blocks in a single climate-controlled room. Four different nutrient loadings (Table 1) were applied to each block of 4 bins to create plant material with a wide range of nutrient contents. Nutrient solutions were made by dissolving NH_4_NO_3_ and KH_2_PO_4_ in deionized water and added to the bins to reach the targeted nutrient loading. Nutrients were added once every two weeks.

**Table 1:** Nutrient addition treatments in the plant culture. Each block had four nutrient addition treatments (N+P+; N-P-; N+P-; N-P+) to maximize differences in plant nutrient composition.

Treatment	N adding (mg L^-1^)	P adding (mg L^-1^)
N+P+	1	0.14
N-P-	0.1	0.014
N+P-	1	0.014
N-P+	0.1	0.14

Each bin was filled with 4 cm sand and filled up with 7 L of tap water. Deionized water was added during the culturing to compensate for evaporation. The climate-controlled room was kept at a constant temperature of 20°C, a day:night cycle of 16:8 h, and light intensity at the water surface was approximately 100 μmol•m^-2^•s^-1^. *Daphnia magna* were introduced to control phytoplankton growth in the water, and a single pulmonate snail *Planorbarius corneus* was added to each bin to control periphyton growth. The snail species does not consume our target plant as determined in pre-trials. The plants were cultured from July 16^th^ to October 1^st^ 2015, after which plant material was harvested from 38 bins that had enough material for feeding trials (at least 0.3 g of fresh leaves was needed for each pair of feeding tests). To increase the power of the experiment, several plants (n = 6) of the treatments which produced a limited amount of plant material provided leaf material for multiple feeding trials.

### Animal food

We chose the crustacean *Gammarus pulex* L. as our animal food source. *G*. *pulex* is one of the most important invertebrate species in temperate streams, and widely distributed throughout Europe [36]. Populations can reach a density of 10000 m^-2^ and it has a continued mortality throughout the year [37]. *G*. *pulex* feeds on a variety of debris, such as oak and elm leaves [38]. Ditches with oak trees along the banks are common in The Netherlands, providing suitable habitat, and there are also plenty of macrophytes and *L*. *stagnalis* in many of these ditches. The habitat of *G*. *pulex* largely overlaps with suitable habitat for *L*. *stagnalis*, as both thrive in macrophyte-dominated ditches and other shallow waters. Therefore, *L*. *stagnalis* can be expected to frequently encounter *G*. *pulex* carrion as possible food source in its natural habitat.

*G*. *pulex* were procured three days before the start of the feeding trial from a ditch close to Wageningen University, The Netherlands (51.989674°N, 5.648653°E). Individuals were placed in continuously aerated groundwater in a plastic tank (0.38 × 0.26 × 0.27 m, L × W × H) and fed detritus from the same ditch. For the experiment, only *G*. *pulex* exceeding 1.4 cm in body size were selected for the feeding trials. Shortly before the feeding trials, *G*. *pulex* were killed in 45°C water before being offered to the snails as snails cannot capture the living *G*. *pulex*, and the *G*. *pulex* would not structurally degrade when killed at this temperature, as was shown from pilot trials.

### Feeding trials

The feeding trials followed standard protocols developed for snails [24, 29, 30]. To test our first hypothesis, we performed feeding trials in which snails were fed plant material of varying nutrient status (the no-choice experiment). To test our second and third hypotheses, we performed feeding trials in which snails were offered both plant and animal food simultaneously (the choice experiment). In total for both experiments, 94 snails were randomly divided into two groups: snails that were to be fed only *P*. *lucens* of varying nutrient status (no-choice plant group, n = 47, replicates for each plant nutrient treatments are: N+P+, n = 12; N-P-, n = 11; N+P-, n = 14; and N-P+, n = 10.), and snails which were offered a choice between *P*. *lucens* and *G*. *pulex* (choice group, n = 47). Additionally, for each plant a portion of the leaves was introduced to a cup without snail, to act as a control (plant control, n = 47). The same was done with *G*. *pulex* (animal food control, n = 12).

Pilot trials demonstrated that a snail consumed a maximum of 0.15g (wet weight) plant food per 24 hours. Leaf portions were sampled as follows: for every plant, all leaves were cut from the stem, including their petiole but excluding stipules. Every leaf had its midrib removed, as this part is not preferred by the snail, and the remaining two halves of lamina were further cut into 6 equally sized pieces, 3 portions for the no-choice plant feeding trial, 2 portions for the choice feeding trial, and 1 portion as plant control. This distribution was expected to minimize or at least randomize the possible differences between leaves within a plant. For the no-choice feeding trials, we therefore weighed approximately 0.15g wet weight plant material from one bin for each snail. For the choice experiments, we combined 0.10g wet weight plant material (from the same bin as the no-choice feeding trial) with 0.19g wet weight animal material for each snail. This is the maximum animal food one snail could eat during 24 h as was tested by pre-trials. For the choice experiment, the amount of plant material and animal food were different, but both were always present in excess amounts for snails to choose from, and the different amounts of food did not affect the selection by pond snails based on pre-trials. We weighed 0.05g plant material and 0.19g animal food for the controls to monitor weight change over 24 h in water without snails. All snails were starved for 48 h before the start of the trials and the feeding lasted for 24 h at 20°C and a dim condition (< 10 μmol m^-2^ s^-1^) with a day:night cycle of 16:8 h. Each snail was fed individually in a plastic cup (top diameter 9 cm, and height 11.5 cm) filled with 375 ml groundwater, covered with a mesh at the top to prevent snail escape.

After all feeding trials, the dry weights of all remaining food as well as uneaten plant material was collected, measured, and dried in an oven at 60°C for over 48 h. All snails were first frozen to death at -20°C, the soft body of the snail was separated from its shell, and then the snail was dried in an oven at 60°C for over 48 h. We measured carbon (C), nitrogen (N), and phosphorus (P) contents of random samples of *G*. *pulex*, n = 12, and *L*. *stagnalis* bodies, n = 13, as well as all 47 plant control portions. Dried samples were ground into fine powders in a 2ml tube on a Tissuelyser II (QIAGEN, Hilden, Germany). C and N contents were determined by an auto elemental analyser (FLASH 2000, Thermo Scientific, Waltham, MA, USA). P content was determined by first incinerating, digesting, and analyzing in an Auto Analyzer (QuAAtro method, Seal Analytical, Fareham, UK).

### Data analyses

Food palatability, represented by food Relative Consumption Rate by the snails (RCR) (mg g^-1^ d^-1^) was calculated by the following formula (after Elger & Barrat-Segretain, 2002):

$$RCR=\left(F_{id}-F_{fd}\right)/S_{d}/1Day$$

Where F_id_ is the initial dry weight of the offered food, F_fd_ is the final dry weight of the retrieved food, and S_d_ is the dry weight of the snail body without shell. To back-calculate the initial dry weight that was offered to the snails from the wet material that was offered, we used extra *G*. *pulex* and *P*. *lucens* leaves to establish dry weight–wet weight regression lines. The regression line for *G*. *pulex* was y = 0.2107*x (*r*^2^ = 0.99, *p* < 0.001, n = 27). For *P*. *lucens* the regression line was y = 0.2477*x (*r*^2^ = 0.97, *p* < 0.001, n = 25), with y giving dry weight in mg, and x being wet weight in mg. Pairwise t-tests showed that the control portion of plant and animal food lost some weight after soaking in the water for 24 h. Plant material average wet weight loss was 0.0019 g (3.8% loss, *t*_46_ = 5.05, *p* < 0.001), and animal food average wet weight loss was 0.021 g (11% loss, *t*_11_ = 7.76, *p* < 0.001). We used this to calibrate the food consumption in the feeding trials by accounting for the lost weight of the control portions of food.

Three snails (2.8%) died during the feeding trials and were excluded from the dataset. During feeding trials in which snails ingested very little material, measurement errors on the wet weight of the offered food can explain why we occasionally report negative plant palatability values. The nutrient treatments in plant culturing were designed for a one-way Anova test, whereas plants did not produce enough leaf biomass for the feeding trials in multiple bins, therefore we decided to analyse the relation between the resulting plant nutrient contents with plant consumption rates using a regression approach. Differences in consumption rates between plant and animal matter were calculated by Students t-tests. Pearson’s correlation was used to test the relation between plant N and P content. One-way ANOVA and Kruskal-Wallis tests were used to test nutrient level differences among the three organisms. Prior to the one-way ANOVA and Student t-tests, Shapiro-Wilk tests were used to confirm the normality of the data. Levene’s tests were used to confirm the homoscedasticity of the data. Non-parametric Kruskal-Wallis tests were used to compare organisms for C content, P content, C:N ratio, C:P ratio and N:P ratio, because not all the assumptions were met. For the linear regressions, we ensured the normality and homoscedasticity of model residuals by plotting quantile-quantile plots and model residuals versus fitted values plots. To test whether plant palatability was affected by plant nutrient status (hypothesis 1), we used linear regressions to relate plant palatability to plant nutrient content and carbon:nutrient ratios of the food. To test for snail diet selection in the choice feeding trials (hypotheses 2 and 3), we calculated the plant material : animal food consumption ratio and used this in linear regression analyses. All statistics were performed in R (R version 3.4.2).

## Results

**Fig 1:**
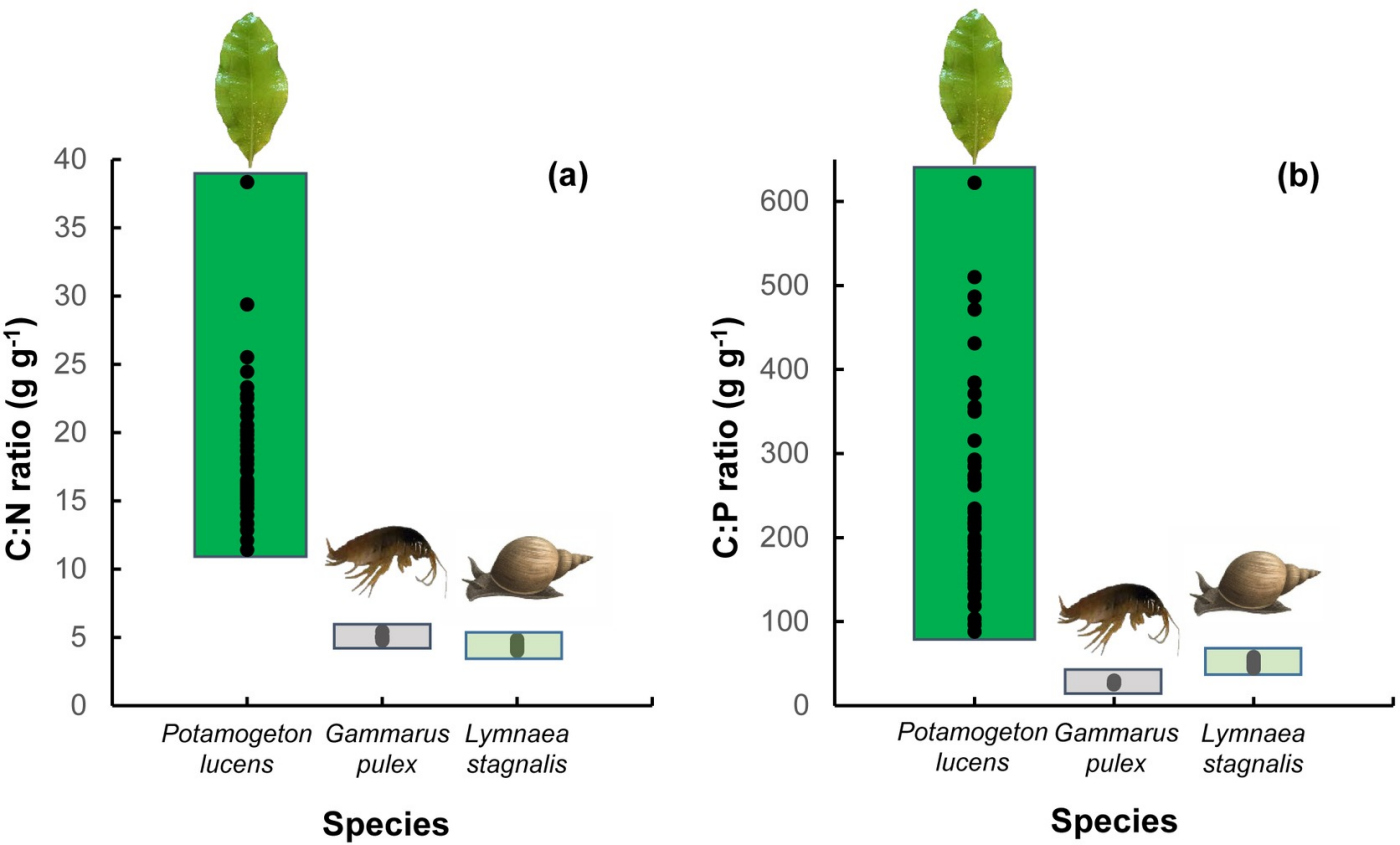
Stoichiometry properties of the organisms used in the study. (a) C:N and (b) C:P stoichiometry for respectively leaves of the plant food *Potamogeton lucens* (n = 47), the animal food *Gammarus pulex* (n = 12) and the omnivorous consumer *Lymnaea stagnalis* (n = 13). Dots in the graph reflect the values measured in the experiment. The plants have been cultured at different nutrient loadings to create a range of plant nutrient contents (see main text).

Plant leaf N content varied from 10.4 mg g^-1^ to 35.7 mg g^-1^, P content varied from 0.6 mg g^-1^ to 4.4 mg g^-1^ and C content varied from 347.7 mg g^-1^ to 407.3 mg g^-1^. Pearson’s correlations showed significant correlations between plant N content and P content (*t*_45_ = 3.55, r = 0.48, *p* < 0.001). The C:N ratio of plant leaves varied over 3-fold (Fig 1A), and the C:P ratio varied over 7-fold (Fig 1B). In contrast, the C:N and C:P ratios of the animal food and the omnivore all varied less than 1.5-fold (Fig 1, Table 2). Organism stoichiometry properties differed among the species, such that the omnivore had a C:N and C:P ratio of its body similar to the animal food. The maximum plant material C:N and C:P ratios were 7-fold higher than the ratios measured for the omnivore and animal prey, whereas the minimum plant material C:N and C:P ratios were only 1.5-fold higher than the ratios measured for the omnivore and animal prey (Fig 1, Table 2).

**Table 2:** Elemental composition and stoichiometry of the study organisms. Different letters in the same column indicate that there is a significant difference among the three organisms. Ratios are presented by first calculating the ratio for each individual data point, and thereafter calculating the means. All numbers are presented as means ± SD.

Type	Species	C (mg g^-1^)	N (mg g^-1^)	P (mg g^-1^)	C:N (g g^-1^)	C:P (g g^-1^)	N:P (g g^-1^)
Omnivore	*L*. *stagnalis* (n = 13)	440.6 ± 6.4^a^	100.6 ± 5.1^a^	8.8 ± 0.8^a^	4.4 ± 0.2^a^	50.2 ± 4.3^a^	11.5 ± 1.0^a^
Animal food	*G*. *pulex* (n = 12)	340.0 ± 8.0^b^	68.4 ± 2.4^b^	12.2 ± 0.5^b^	5.0 ± 0.2^a^	27.8 ± 1.4^b^	5.6 ± 0.3^b^
Plant material	*P*. *lucens* (n = 47)	390.4 ± 11.6^c^	22.9 ± 5.1^c^	1.9 ± 0.9^c^	18.0 ± 4.8^b^	247.2 ± 120.3^c^	14.0 ± 6.5^a^
Plants in four nutrient treatments	N+P+ (n = 12)	393.6 ± 5.8	24.9 ± 4.9	2.7 ± 0.7	16.5 ± 4.3	159.6 ± 47.2	9.7 ± 1.7
	N-P- (n = 11)	388.2 ± 8.9	20.4 ± 3.9	1.6 ± 0.3	19.5 ± 3.0	247.9 ± 57.1	12.9 ± 3.3
	N+P- (n = 14)	396.7 ± 5.1	24.2 ± 2.5	1.2 ± 0.4	16.5 ± 1.6	374.7 ± 125.4	22.3 ± 5.5
	N-P+ (n = 10)	380.3 ± 18	21.6 ± 7.7	2.5 ± 0.9	20.0 ± 8.1	177.0 ± 84.1	8.8 ± 1.6

**Fig 2:**
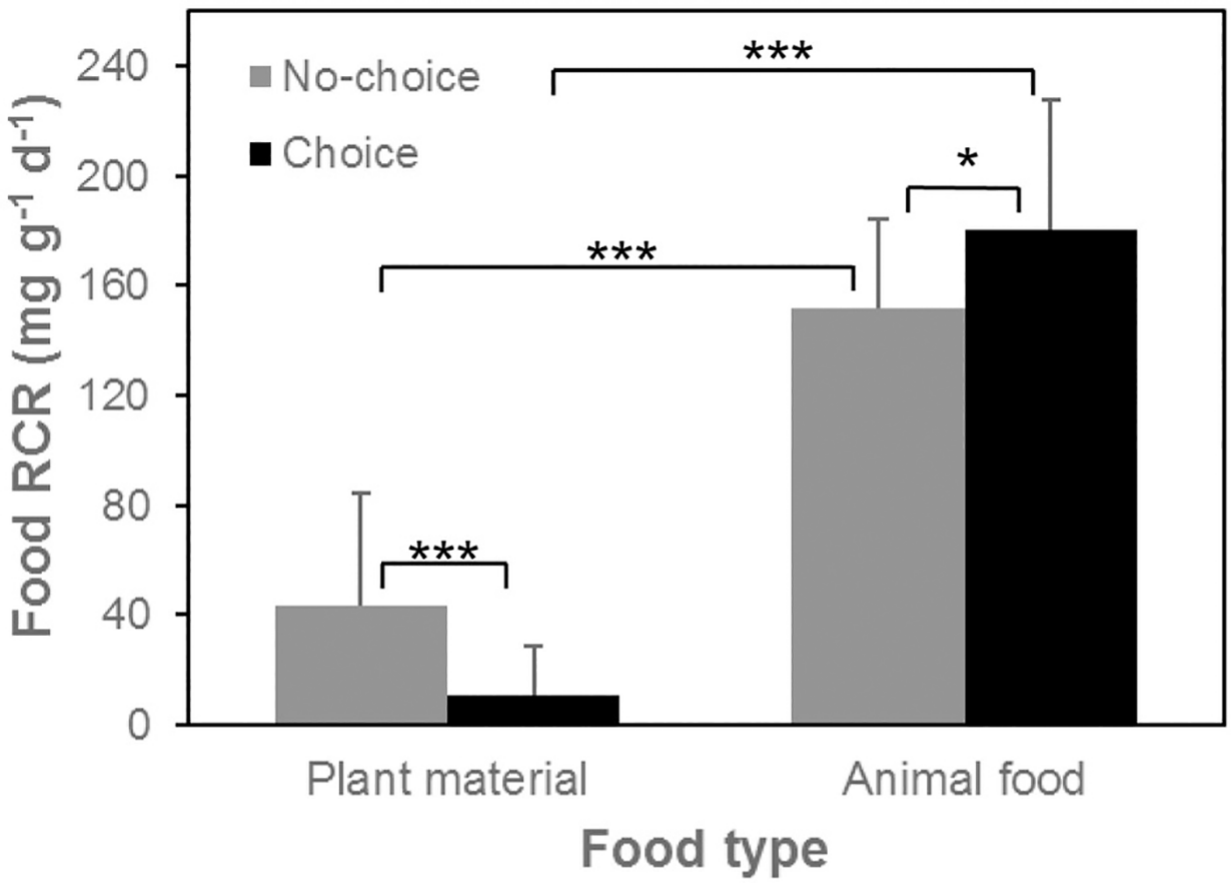
Relative consumption rates of plant material and animal food by the omnivore *L*. *stagnalis* in both choice and no-choice trials. RCR indicates relative consumption rates in mg dry weight consumed per gram dry weight snail body mass per day. * indicates *p* < 0.05, and *** indicates *p* < 0.001.

**Fig 3:**
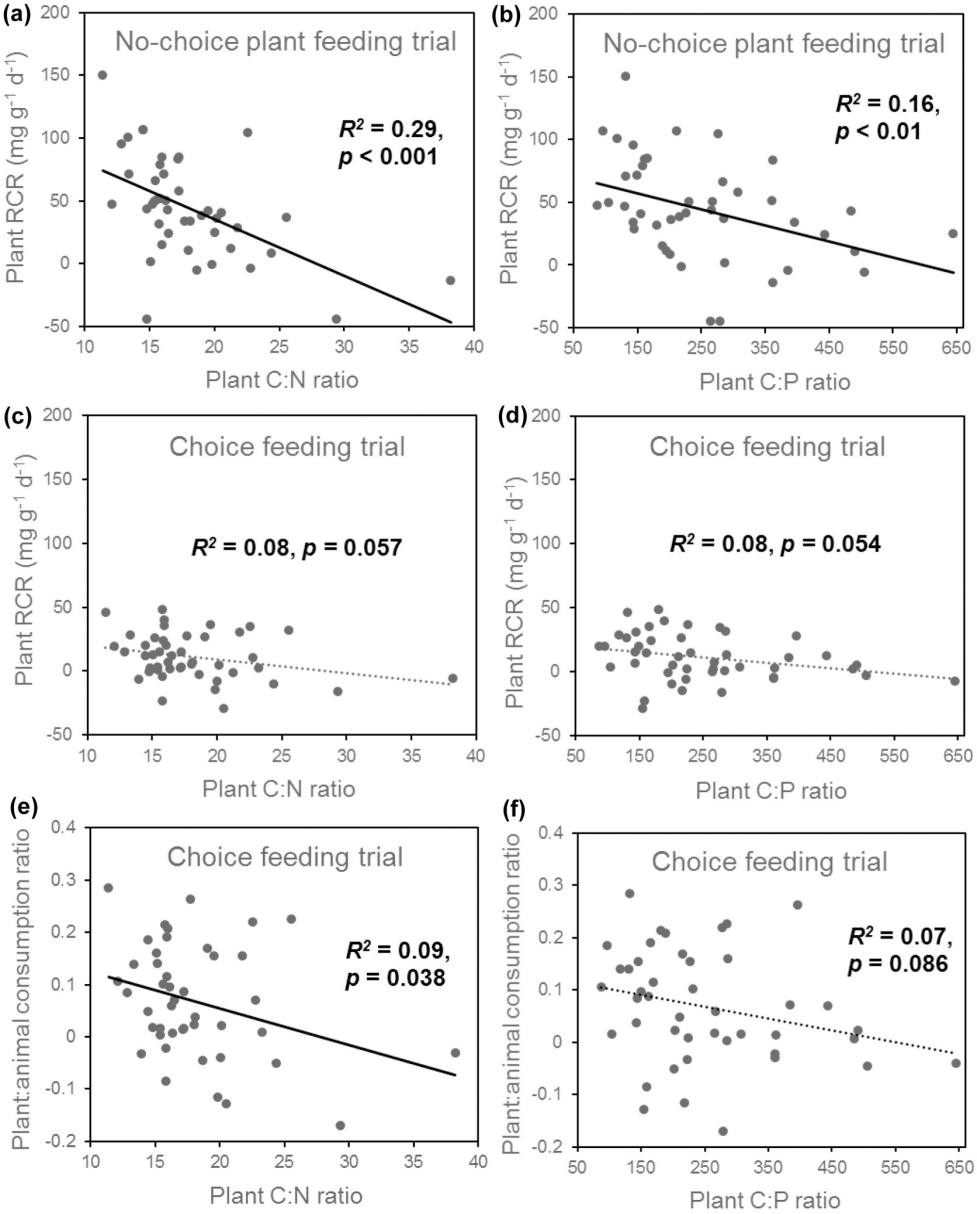
Plant relative consumption rates (RCR) correlations with plant C:N and C:P ratios in both choice and no-choice feeding trials. Plant relative consumption rate in no-choice trials decreased with the increase of the plant C:N ratio (a) and plant C:P ratio (b). Plant relative consumption rates in choice trials decreased with the increase of the plant C:N ratio (c) and C:P ratio (d). Plant material : Animal food consumption ratio in choice trials decreased with the increase of the plant C:N ratio (e) and C:P ratio (f). Solid regression lines indicate *p* < 0.05 and dotted lines indicate 0.05 < *p* < 0.1.

Plant material consumption rates were higher in the no-choice trial than in choice trial (in the no-choice trial, 43.4 ± 40.7 mg g^-1^ d^-1^; in the choice trial, 11.0 ± 18 mg g^-1^ d^-1^, mean ± SD, t-test, *t*_60_ = 4.89, *p* < 0.001, Fig 2). However, animal food consumption rates were lower in the no-choice trial than in the choice trial (in no-choice, 151.7 ± 32.5 mg g^-1^ d^-1^; in choice, 180.4 ± 47.7 mg g^-1^ d^-1^, mean ± SD, t-test, *t*_25_ = -2.44, *p* = 0.02, Fig 2). In the no-choice feeding trial, plant relative consumption rates by snails increased with increasing plant N content (*F*_1,42_ = 19.93, *p* < 0.001) and plant P content (*F*_1,42_ = 11.77, *p* < 0.01), and decreased with increasing of C:N ratio (*F*_1,42_ = 16.76, *p* < 0.001, Fig 3A) and C:P ratio (*F*_1,42_ = 8.101, *p* < 0.01, Fig 3B). In the choice feeding trials with both plant and animal matter present, animal material was consumed in much larger quantities than plant material (pairwise t-test, *t*_45_ = -26.56, *p* < 0.001, Fig 2). Animal consumption rate was not affected by plant N content (*F*_1,44_ = 0.44, *p* = 0.53), plant P content (*F*_1,44_ = 0.66, *p* = 0.42), plant C:N ratio (*F*_1,44_ = 0.92, *p* = 0.36, Fig 3), nor plant C:P ratio (*F*_1,44_ = 1.21, *p* = 0.28, Fig 3). In the choice trials, plant consumption rate shows a strong increasing trend as plant N content increased (*F*_1,44_ = 3.90, *p* = 0.06) and as P content increased (*F*_1,44_ = 3.60, *p* = 0.06), as C:N ratio decreased (*F*_1,44_ = 3.97, *p* = 0.057, Fig 3C) and as C:P ratio decreased (*F*_1,44_ = 3.93, *p* = 0.054, Fig 3D). The plant:animal food consumption ratio in the choice feeding trials increased as plant N content increased (*F*_1,44_ = 4.72, *p* = 0.035) and as plant C:N ratio decreased (*F*_1,44_ = 4.58, *p* = 0.038, Fig 3E). The plant:animal food consumption ratio also showed an increasing trend as plant P content increased (*F*_1,44_ = 3.38, *p* = 0.071) and as plant C:P ratio decreased (*F*_1,44_ = 3.09, *p* = 0.086, Fig 3F).

## Discussion

In this study we experimentally tested whether aquatic omnivores increase plant consumption as plant quality, expressed as nutrient content and C:nutrient stoichiometry, increases. We found that when there was only plant material available, the omnivores increased plant consumption as plant quality increased from no measurable amount to about 102 mg g^-1^ day^-1^. When plant material was offered simultaneously with animal food, the omnivores strongly preferred animal food, a result which mirrored the intake rates of plant and animal food in the no-choice tests. Despite this preference for animal food, the omnivores increased their relative intake of plant material from virtually nothing at the lowest plant quality to on average 16% (maximum 28%) of their diet at the highest plant quality. We conclude that as plants increase nutrient content during increasingly frequent nutrient loading in aquatic systems, omnivorous animals may shift their trophic position resulting in increased top-down control on aquatic plants.

When there was only plant material available, the omnivore consumed more plant material as the plant C:nutrient ratio decreased. Hence, this supports our first hypothesis. Generally, this is a classic observation that herbivore removal of plant standing biomass increases as plant N content increases [22, 39, 40]. The increased consumption might be more than the re-growth of the plant, leading to enhanced top-down control on plant standing biomass. This has been demonstrated by fertilization experiments with mallard duck [12], and is also supported by modelling studies [13, 41]. Higher consumption rates in no-choice feeding trials have also been interpreted as compensatory feeding, where the consumer needs to feed more on a poor quality resource to meet its nutrient or energy demands [42, 43]. However, by comparing our no-choice and choice feeding trials we can demonstrate that this is not the case in our experiment. The snails ate 4-fold more animal food compared to plant food in 24 hour no-choice feeding trials, whereas plants were of lesser quality than animal food. Hence for compensatory feeding they should have eaten much more plant food to compensate for the low nutrient levels in plant food. Furthermore, the outcome of the no-choice tests reflected those of the choice tests very well, where the snails similarly consumed much more animal food. When there was animal food available, the omnivore always showed a much stronger preference for animal food, thus confirming our second hypothesis. Yet, the snails showed an increased preference for plant material as the plant C:nutrient ratio decreased, supporting our third hypothesis.

As the plant nutrient content increased and the C:nutrient ratio decreased, the quality difference compared to animal food decreased, thus making plant food relatively more attractive. In our study plant consumption increased from about zero at low plant quality to a maximum of 28%. This indicates that the snail was still highly omnivorous, with a preference for animal food, but does include a substantial amount of plant food in its diet. These results are in line with the notion that most of the generalist feeders try to consume a mixed diet to balance their nutrition intake [31, 44–46]. Food searching is a very cost-intensive process for the snails due to their low motility. In order to maximize the fitness of the feeding [47], the snails include relatively more plant material in their diet as plant quality is getting closer to their nutrient demand. Similarly, omnivorous fish [48, 49] and mallard duck [12], increased plant consumption with increasing plant quality, or shifted their diet to alternative prey when aquatic plants were not palatable. Furthermore, the no-choice feeding trials demonstrate that when the snails have no animal food available they ingest even more plant material. Therefore, with eutrophication, aquatic plants are likely to have more top-down pressure, from both aquatic herbivores and omnivores.

In this study, we employed nutrient contents and C:nutrient stoichiometry as a proxy for plant quality for omnivores. The C:nutrient stoichiometry of food has been shown to be a good indicator of food quality to aquatic animals, where a lower C:nutrient ratio represents higher quality [10, 17, 21]. Even though plant secondary metabolites might deter animals from feeding on the plant [21, 50], this is not the case for *P*. *lucens*, as it contains very low total phenolic concentrations compared to other aquatic plants [24]. Furthermore, the concentration of plant phenolic compounds might decrease as plant nutrient content increases, as has been shown in seagrass [51]. *P*. *lucens* is a moderately palatable aquatic plant species based on palatability rankings among a wide range of species from Elger et al. [33] and Grutters et al. [24], which indicates that this species can well represent many other aquatic plant species. In our study, the variation in plant C:nutrient ratios was much larger than the variation in C:nutrient ratio of the animal food and the omnivore. The reason might be that the sample size was larger for plants than the animals that we tested and the plants received different nutrient addition treatments. However, generally, plants do have a much broader range of C:nutrient ratios than animals [17, 19]. Recent studies show that the C:nutrient stoichiometry of aquatic invertebrates can also vary in eutrophic conditions [52]. However, with C:N ratios varying from 3.8 to 7.7 g g^-1^ [52], the variation is still much smaller than the C:N ratio of the plants in our study and the C:N ratio in other aquatic plants [10]. Therefore, our study still has implications for aquatic plant-omnivore interactions in general.

### Implication for ecosystems

In shallow aquatic ecosystems, the growth of aquatic plants is also inhibited by shading of phytoplankton and periphyton [1, 41, 53]. There is a pervasive top-down pathway through which omnivores can influence aquatic plants from omnivores (fish and birds) to invertebrates (both zooplankton and macroinvertebrates) to algae (both phytoplankton and periphyton) to aquatic plants. The omnivores can inhibit the growth of aquatic plants indirectly by feeding on invertebrates, which graze on algae, thereby releasing the algae from grazing pressure and subsequently, the algae can inhibit the growth of aquatic plants [1, 41, 53–55]. With eutrophication, positive feedback exists where increased primary producer abundance leads to increased omnivore abundance and pressure on invertebrates, which results in less grazing pressure on algae and more shading of aquatic plants [41, 56]. A similar phenomenon has also been observed in terrestrial ecosystems where increased plant quality can stabilize an omnivore population, and keep the pest animal prey at a low level [25]. On the other hand, some aquatic omnivores can also directly affect aquatic plants by consuming them [12, 41, 56]. Here, plant quality can increase with eutrophication and the omnivores increase their consumption of aquatic plants. In addition, if the animal prey is not available, the omnivore might feed more on aquatic plants. Therefore, under eutrophication, omnivores are expected to impose a stronger top-down control on aquatic plant standing biomass both indirectly by increasing the shading pressure by algae and directly by increased plant consumption [41]. While the former mechanism has been well documented, the latter has largely been overlooked. With this study, we demonstrate that omnivores increase their impact on aquatic plants under eutrophication by shifting their trophic position towards enhanced plant consumption. The combined stress of shading by algae and grazing pressure by omnivores and herbivores under eutrophication can lead to disappearance of submerged aquatic vegetation and a shift to a turbid state dominated by phytoplankton [13, 41].
